# The relationship between labial soft tissue changes and jumping spaces after immediate implant placement and restoration in the anterior maxilla: A prospective study

**DOI:** 10.17305/bb.2023.8652

**Published:** 2023-10-01

**Authors:** Linkun Xu, Sui Zhang, Yue Chen, Feiyan Yu, Chong Han, Dongchao Wu, Dongning He

**Affiliations:** 1 Department of Implantology, Shanxi Medical University School and Hospital of Stomatology, Taiyuan, China; 2 Department of Oral Implantology, Xiangyang Stomatological Hospital, Xiangyang, China; 3 Shanxi Medical University School and Hospital of Stomatology, Taiyuan, China

**Keywords:** Immediate implant insertion/placement, jumping space (jumping gap), immediate provisionalization, soft tissue, esthetic region

## Abstract

Oral implants have been increasingly used in the treatment of edentulous patients or those with dentition defects due to reliable treatment procedure and favorable long-term prognosis. We investigated the changes of labial soft tissue contours with different jumping spaces after immediate implant placement and restoration (IIPR) in the maxillary esthetic area and also provided a long-term stability measurement for the changing trend of soft tissue contour. All patients had been separated into three groups based on the jumping space: group A (horizontal defect dimension [HDD] ≤ 2 mm), group B (2 mm < HDD ≤ 3 mm), and group C (HDD > 3 mm) and the digital impressions were obtained in the first, third, and sixth month after the operation. The changes of gingival mucosa levels, the average thickness of soft tissue contour volume, and the linear change of submarginal level decreased gradually across the three groups, with the largest change of submarginal level being at 5 mm. The size of the jumping space was moderately negatively correlated with the level and average thickness of gingival mucosa and the linear changes of 3 mm and 5 mm under gingival margin, while there was no significant correlation with pink esthetic score (PES) and the linear change of the 1 mm under the gingival margin. Generally, IIPR of upper anterior teeth can achieve esthetic satisfaction, and the level of soft tissue around the implant can be well preserved.

## Introduction

Since Professor Brånemark discovered osseointegration in 1962 and provided the world’s first pathological tissue grinding film in 1981, oral implants have been utilized in increasing number of edentulous patients or patients with dentition defects due to their highly predictable treatment procedure and good long-term prognosis [1]. In the traditional procedure, endosseous implants require three to six months to fully integrate with the underlying bone before implants can be loaded [2]. The present studies have demonstrated that it is easier to obtain good initial stability in the sockets that have been filled with new bone after three to six months following tooth extraction [3] but patients’ satisfaction from this procedure reduced as the total treatment time increased. A study conducted by Gotfredsen et al. [4] in 2021 concluded that patients preferred shorter-time treatments to traditional delayed implants, especially in the upper anterior esthetic zone. Presently, much literature shows that immediate implantation at the newly created extraction sockets, namely, immediate implant placement (IIP), has been suggested to shorten the prosthetic treatment time [5].

In 1976, the insertion of implants into newly created extraction sockets, the so-called ‘Turbingen implant,’ was first described by Schulte and Heimke [6]. An ideal of three-dimensional (3D) implant positioning, fewer surgical interventions, the shortening of the overall treatment time, the putative retention of alveolar tooth structure at the side of sockets and improved soft tissue aesthetics have been touted as having potential benefits of the IIP treatment approach [7]. However, IIP also has its inherent defects. In general, it is more difficult to achieve the initial closure of soft tissue than with other types because of the lack of keratinized mucosa in the surgical area. Moreover, the inconsistency between both size and shape of the extraction sockets and the implant is detrimental to the initial stability because the dental implant must be in intimate contact with the alveolar bone in order to be retained [8].

A systematic review [9] analyzing dimensional alteration in the alveolar ridge revealed that the horizontal bone loss was more significant than the vertical bone loss (29%–63%, 3.79 ± 0.23 mm vs 11%–22%, 1.24 ± 0.11 mm) at the sixth month after tooth extraction. Most of the decrease in alveolar bone occurs within three to six months after extraction and reduces gradually. Some scholars speculate that direct implantation into fresh extraction sockets before bone resorption may be able to preserve the alveolar bone around the natural teeth. However, existing animal experiments and clinical trials have shown that IIP fails to avoid the alveolar ridge’s resorption, especially the alveolar crest on the labial side [10, 11]. Also, even though longitudinal studies typically claimed that dental implants have a success rate of up to 90%–95%, the gingival recession is frequently reported as a complication, in up to 40% of cases [12].

Many studies have determined the relevant factors affecting bone resorption following immediate implantation [13], including the implant size, shape of the alveolar ridge, buccal bone plate thickness [14, 15], application of bone graft material, and soft tissue transplantation [16]. The 6th EAO Consensus Conference pointed out that immediate implant placement has a high survival rate [17]. Also, IIP has been widely used in daily clinical operations. However, as mentioned earlier, it is difficult to close the wound after the procedure. Therefore, immediate restoration while simultaneously closing the wound becomes an ideal method [18]. The appearance of patients can be restored immediately, therefore, it is especially favored by patients in clinics [19].

Immediate implant placement and restoration (IIPR) is also a successful technique that can achieve similar results to many other treatment techniques [20] and improves the esthetic effect and bone level around the implant in patients with a temporary prosthesis [21]. However, due to the difference between the extraction socket’s size and the implant’s diameter, when IIPR is performed at the upper anterior tooth area, placing the implant into the extraction socket will inevitably lead to a horizontal defect dimension (HDD) on the implant’s labial side, which is also known as jumping space/jumping gap [22, 23]. Although there was a clinical report suggesting that spontaneous bone filling had a high percentage for direct implantation when the marginal bone-implant gap was greater than 2 mm [24], studies suggested that the use of bone grafts to fill the gap may help reduce bone resorption and maintain the appearance and esthetics of the soft tissue [25, 26]. In particular, the soft tissue’s volume profile around the anterior region is extremely significant [27]. So, there is a question if the size of the jumping space will affect the change of gingival volume profile after IIPR. Regarding the type of restoration that can be supported on post-extraction implants, screw-retained restorations are always preferred due to the often-present particularly high mucous cones or soft tissue volumes with unpredictable amplitudes at the time of implant placement, and above all during the delivery of the provisional prosthetic restoration [28]. The initial insertion of the implant has little effect on the resorption of the coronal marginal bone, but has a significant effect on the soft tissue volumes if properly provisioned. This renders peri-implant soft tissue augmentation operations significantly less common and unnecessary [29]. Therefore, it is necessary to further observe and measure the differences in the gingival soft tissue volume profile of different jumping spaces during IIPR. However, the measurement of soft tissue volume is an intricate challenge, especially the observation of its dynamic changes [30]. With the invention of the digital 3D optical scanning image technique, some scholars have introduced it to evaluate and measure the volume change of oral soft tissue over time [31]. This method provides a new way for direct measurement and 3D display of soft tissue volume. Besides that studies have demonstrated that this non-invasive method has high accuracy and reliability in evaluating the changes in soft tissue volume [32].

In this study, this repeatable measurement method was used to evaluate the dynamic changes of labial gingival soft tissue contours in different jumping spaces of IIPR in the upper anterior area to provide a basis for clinical evaluation of the changing trend and stability of labial soft tissue contours in patients with different jumping spaces.

## Materials and methods

### Study participants

To achieve the research objective, the authors planned and conducted a prospective study. The study population consisted of all patients presenting for evaluation and IIPR treatment between March 2020 and September 2021. This study included 32 patients who had a single tooth in the upper anterior region and required IIPR at the Department of Implant, Stomatological Hospital of Shanxi Medical University. The sample size was calculated by using the following formula:

$$n=Z^{2}p(1-p)⁄m^{2}$$

*n* ═ sample size, *Z* ═ z value, *m* ═ margin of error, and *p* ═ proportion of population.

All patients who met the inclusion criteria were informed about the clinical procedures, substitute therapeutic methods, and potential risks and complications of this study, and signed an informed consent form before the procedure. All patients (mean age 31.1 ± 7.5 years old) were treated with the same surgical and prosthetic procedure by the same senior implant specialist, and the intraoral scan was performed by the same technician before the operation (baseline) and in the first month (T1), third month (T3), and sixth month (T6) after the procedure. An experienced operator obtained digital impressions using an intraoral scanner (3Shape Trios, 3Shape, Denmark; software version: 2014-1) preoperatively (baseline), in the third month, and sixth month before delivery of the definitive restoration, all with the same scanning strategy.

### Inclusion criteria

Patients requiring extraction of a single maxillary anterior tooth (central incisor, lateral incisor, or canine) must have had adjacent teeth on both sides; be 18 years of age or older; and with good oral hygiene (after basic periodontal treatment, i.e., scaling and root planning). Plaque scores were recorded at 3, 6, and 12 months for the implant repair and the neighboring tooth. Four sites were scored on a dichotomous scale (where zero indicated no apparent plaque at the soft tissue margin and one indicated obvious plaque at the soft tissue margin [mesial, midfacial, distal, and palatal]); Using a manual probe, the probing depth (PD) and bleeding on probing (BOP) at mesial, midfacial, distal, and palatal locations were measured in order to assess the peri-implant health of the implant restoration after 12 months (CP 15 UNC, Hu-Friedy, Chicago, Illinois). All PD measurements were taken to the nearest 0.5 mm, and BOP was scored on a binary scale (zero for no bleeding; one for bleeding); minimum width of keratinized gingiva was ≥ 1 mm. Patients also had to meet the following indications for IIPR: complete alveolar fossa, no acute inflammation in the roof of the implant site, the bone mass of the root of the affected tooth of 3–5 mm to ensure initial stability (≥ 35 Ncm), the stable patients’ occlusal relationship, that the patients could be revisited according to the appointment, and that the informed consent form for the procedure was signed.

### Exclusion criteria

Exclusion criteria included patients with poor general health (e.g., history of head and neck radiation, history of bisphosphate treatment, uncontrolled diabetes, etc.), poor lifestyle habits (e.g., bruxism, heavy smokers [>10 cigarettes/day]), patients with extremely poor oral hygiene condition, those who had acute infection in the apical region of the affected teeth and periodontal soft and hard tissue inflammation in an acute active stage (patients with pus or fistula), patients in whom the implant did not have good initial stability after surgery (the implant torque was less than 35 Ncm) and patients whose teeth could not be repaired immediately.

### Materials and instruments

Following materials and instruments have been used in this study: Nikon D90 SLR camera (Nikon Co., Ltd., Japan), Trios 3 (3Shape, Denmark), CBCT (KaVo, Germany), Geomagic Studio 2013 (Geomagic, USA), Geomagic qualify 2013 (Geomagic, USA), Minimally invasive elevator (Luxator, Sweden), Ankylos implant (Dentsply Sirona, Germany), Nobel Active implant and temporary titanium abutment (Nobel Biocare, Sweden), Bio-oss bone powder (Geistlich Pharma AG, Switzerland), 3M Z350 flow resin (3M Company, USA), DMG Luxatemp star temporary crown material (DMG, Germany), 1.7 mL × 1 dose of articaine epinephrine injection (ACTEON, France), Amoxicillin capsules 0.5 g × 24 tablets (Federal Pharmaceutical Company, China), Roxithromycin 0.15 g × 12 tablets (Zhenyuan Pharmaceutical Co., China), Tinidazole 0.5 g × 8 tablets (Hubei Guangji Pharmaceutical Co., Ltd., China), Celecoxib capsules 0.2 g × 6 tablets (Pfizer, USA), Ibuprofen sustained release capsules 0.3 g × 20 tablets (GlaxoSmithKline, UK), and Compound chlorhexidine gargle 500 mL (Jiangsu Chenpai Bond Pharmaceutical Co., Ltd., China).

### Clinical procedures

Complete preoperative evaluation was carried out, including periodontal examination, clinical photography, smoking habits, necessary imaging diagnosis, assessment of the alveolar ridge’s shape in the implant area, the incisive canal’s location, the affected teeth to be extracted, the bone mass of the root area, and measurement of the labial bone plate’s thickness at the implant site. Light smokers were prohibited from smoking one week before and one month after operation. Patients would need to take the diagnostic model, arrange the wax teeth and take the local silicone rubber impression in the wax tooth area to make the silicone rubber guide plate, which was expected to direct the adjustment and modification of the temporary abutment and the manufacture of the temporary crown.

Before the operation, patients were advised to follow the management of gingivitis, i.e., root planning and scaling were done for all the patients prior to the implant surgery following the above-mentioned protocol. Before any implant procedures were performed, prophylactic antibiotic medication was administered, such as taking 0.5 g amoxicillin capsule (0.15 g roxithromycin for patients with amoxicillin allergy), 0.5 g tinidazole tablet; 0.2 g celecoxib was administered (0.3 g ibuprofen sustained-release capsule for allergic patients), and oral disinfection was performed (solution of 0.2% chlorhexidine for 1 min).

Platelet-rich fibrin (PRF) preparation was straightforward and used the same equipment as platelet-rich plasma (PRP). Two 6 mL sterile vacutainer tubes without anticoagulant were used for collecting 5 mL of whole venous blood (10 mL). After 10 min in a centrifugal machine at 3000 rpm, the vacutainer tubes settled into three layers: red lower fraction with red blood cells, top straw-colored cellular plasma, and middle fraction with fibrin clot. After removing the upper straw-colored layer, the PRF as the intermediate portion was collected 2 mm below the lower dividing line. Due to centrifugation, fibrinogen from the high part of the tube mixed with circulating thrombin to produce fibrin. A fibrin clot was then formed in the tube between the red corpuscles at the bottom and the acellular plasma at the top. Many platelets were caught by fibrin meshes.

The surgical area was prepared and draped in a standard surgical procedure, and then intraoral local anesthesia (articaine 4%) was administered. A minimally invasive elevator was used to remove the affected teeth (splitting labiolingually if necessary) to protect the bone wall of the extraction fossa, especially the bone wall on the labial side, and the integrity of the extraction socket was inspected consequently. The labial bone wall of the extraction fossa included in this study had to be intact with no defects or perforations, otherwise, flap surgery and bone augmentation were performed and those patients were excluded.

The implant site was drilled sequentially in the palatal position of the extraction fossa, and the direction aimed to obtain the retention of the apical and palatal bone plate without breaking through the plate. Based on the density of the remaining bone, the diameter of the implant site had to be at least 1 mm smaller than that of the implant. Two types of implants, Nobel Active and Ankylos (diameter: 3.5 mm, length: ≥ 13 mm), had been used in this study to ensure that the retention provided by the root was greater than or equal to 35 Ncm.

The correct 3D position of the implant is extraordinarily vital, and simultaneously the adjacent teeth were used as a reference to obtain the ideal position of the implant. In all cases, the cervical platform of the implant showed a minimum of 1.5 mm distance from the adjacent teeth, while the implant was approximately 3–4 mm below the gingival margin.

The titanium temporary abutment was connected intraorally (the carrier of the Ankylos implant can be used as the temporary abutment). Under the guidance of the silicone rubber guide plate, the temporary abutment was adjusted to reserve enough space for the temporary crown made by DMG temporary crown material intraorally. The temporary crown was removed after the material was cured and the gingival part was modified with 3M Z350 flow resin and strictly polished and sterilized.

**Figure 1.**
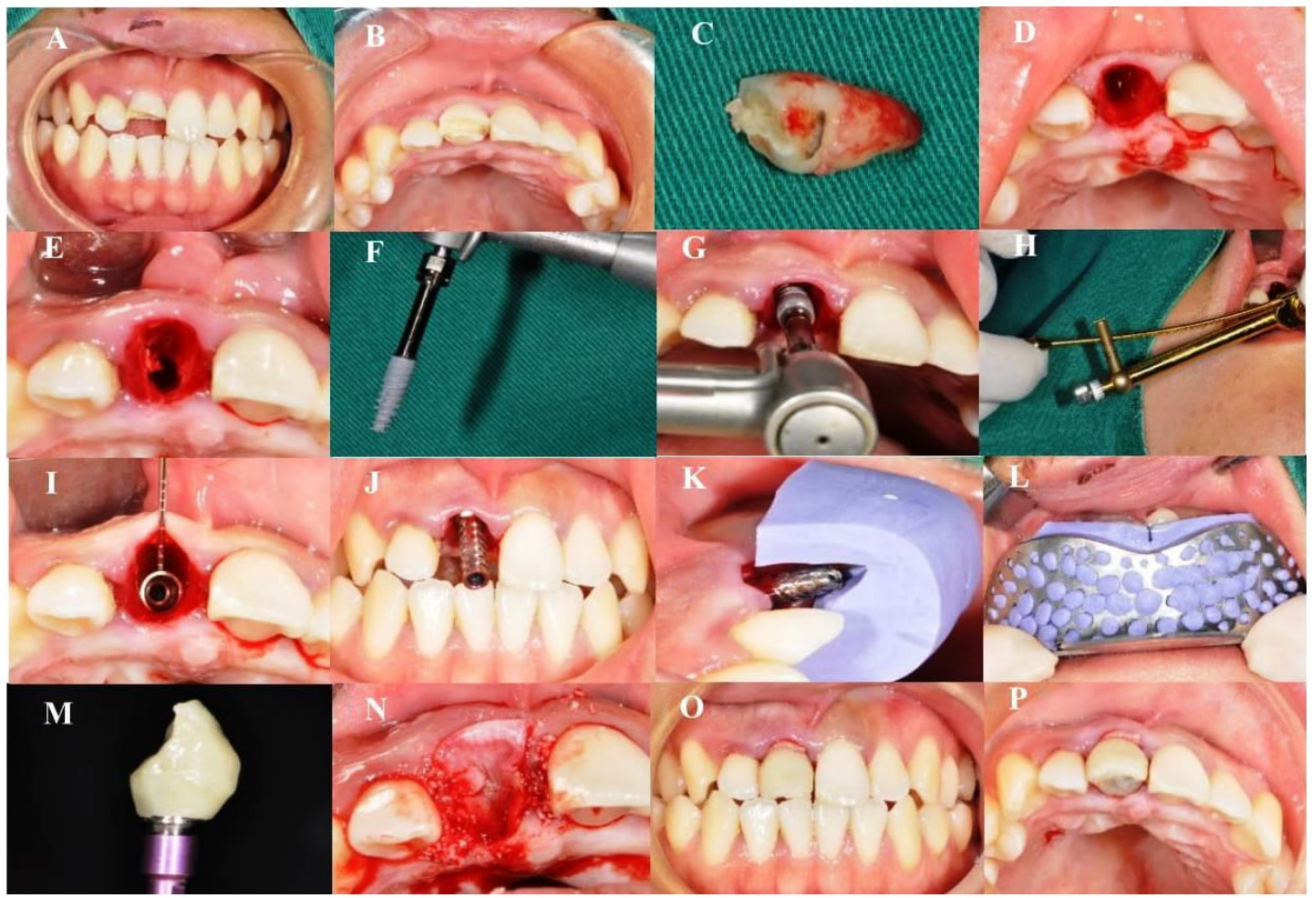
**Clinical procedure.** (A and B) Frontal and occlusal photos before the operation; (C and D) Minimally invasive extraction of the affected teeth; (E) Preparation of holes in the palatine side of the alveolar fossa; (F–H) Implantation of Nobel Active 3.5 × 13 mm with implant torque > 40 Ncm; (I) Measurement of jumping space; (J) Connection of temporary abutment; (K and L) Silicone rubber guide plate down grinding temporary abutment; (M) Restored temporary crown; (N) Bio-oss bone powder implanted in the jump space and covered with platelet-rich fibrin (PRF) film; (O and P) Frontal and occlusal photos after the operation.

A small amount of low replacement bone substitute material (Bio-oss bone powder) that was mixed with autologous blood was filled into the jumping space many times to ensure dense filling in the space. Then, the PRF film was covered and the adjusted screw retention temporary crown was worn without contact with the opposite jaw teeth during protrusion and lateral movement (Figure 1). Cone-beam computed tomography (CBCT) images were used to measure the distance between the labial bone plate and the surface of the implant, i.e., the width of the jumping space. After the surgery, patients were advised to take antibiotics and painkillers. Patients were instructed to ice compress in the operation area for 6 h and gargled with 0.12% chlorhexidine solution 24 h after the procedure for 10 days, 3 times a day.

**Figure 2.**
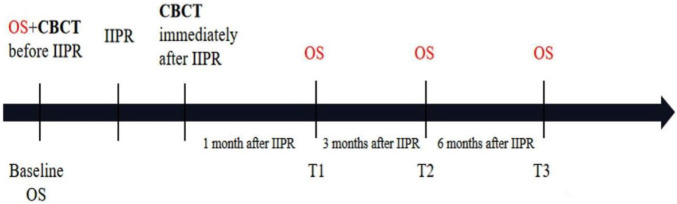
**Treatment procedure and observation time points.** OS: Oral scan; CBCT: Cone-beam computed tomography; IIPR: Immediate implant placement and restoration. T1: Month 1 after the operation; T2: Month 3 after the operation; T3: Month 6 after the operation.

### Data acquisition and follow-up

**Figure 3.**
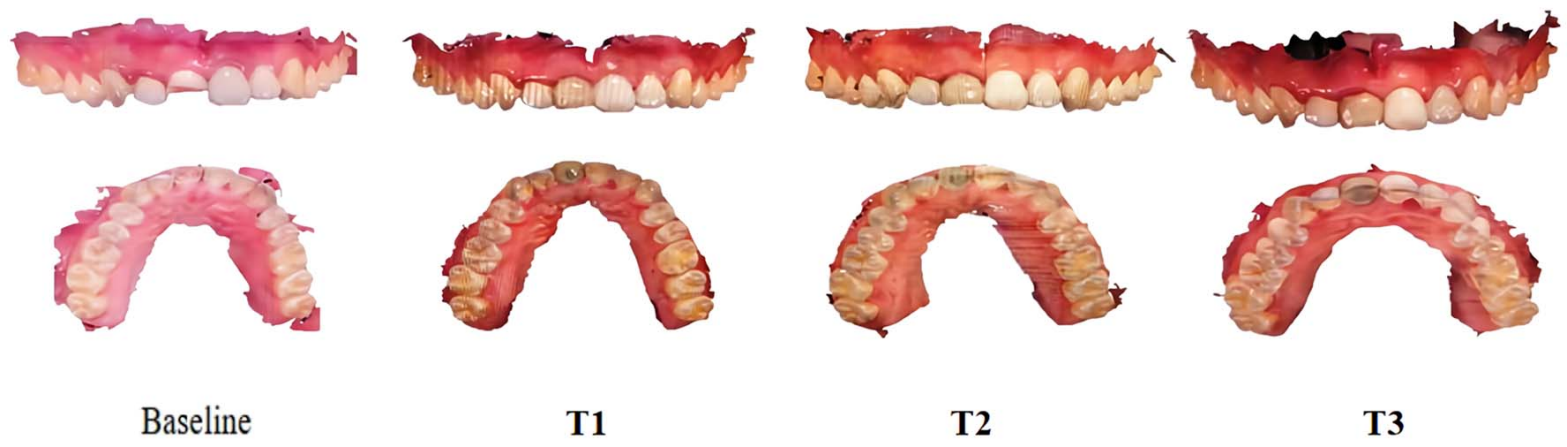
**Oral scan model at every time point.** All patients were treated with the same surgical and prosthetic procedure by the same senior implant specialist, and the intraoral scan was performed by the same technician before the operation (baseline) and in the first month (T1), third month (T2), and sixth month (T3) after the procedure. An experienced operator obtained digital impressions using an intraoral scanner (3Shape Trios, 3Shape, Denmark; software version: 2014-1) preoperatively (baseline), at T1, and T3 before delivery of the definitive restoration, all with the same scanning strategy.

Before the procedure, the maxillary dentition of all patients was scanned by the same senior technician in Trios (3Shape) as the baseline. During the follow-up period (Figure 2), all patients received requisite oral hygiene maintenance, intraoral photos, and necessary radiographs. The scanning range included maxillary complete dentition and labial gingival soft tissue structure for later clipping and registration; obtained digital impressions were saved and exported in the Standard Tessellation Language (STL) format (Figure 3).

The thickness of both labial bone plate of the implant site and the labial bone wall of the implant which is the difference in jumping spaces size was measured by the same radiologist with CBCT (KaVo, Germany) immediately after the operation. Particularly, the shooting conditions of CBCT were constant (voltage 120 kV, current 30 mAS) and the images were imported into eXamVision software in DICOM format for measurement. In order to select the matched section, we found the same reference point in the image software (such as the 3D position of the adjacent tooth or the contralateral control tooth) and obtained the matched section by switching the sagittal plane layer by layer carefully.

**Figure 4.**
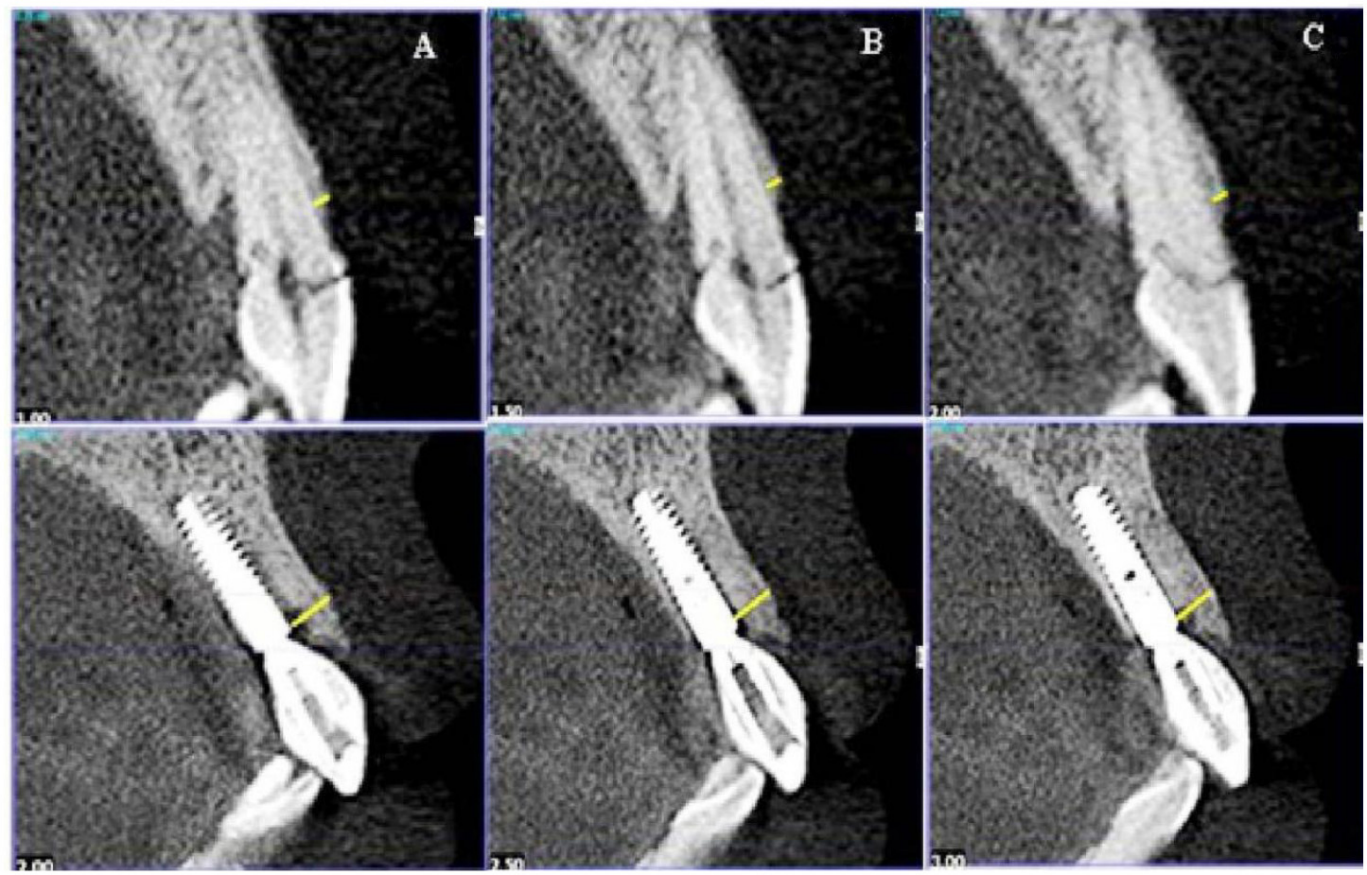
**Measurement and calculation method of jumping space.** Three sagittal planes (adjacent 1 mm) were required for the labial bone plate’s thickness measurement before and after the operation, and the average value of the three measurement points was taken as the difference between the two points: (A) Mesial section; (B) Median section; (C) Distal section.

The labial bone plate’s thickness of the affected teeth was measured at the median sagittal plane, the mesial 1 mm sagittal plane, and the distal 1 mm sagittal plane of the labial bone plate to the 1 mm alveolar crest. The average value of the three measuring points was recorded as the thickness of the labial bone plate (W1). During operation, the implant is usually embedded at the subgingival margin of 3–4 mm, which is approximately the same as the 1 mm below the alveolar crest. Therefore, the measurement of the thickness of the labial bone plate had been taken at the shoulder of the implant, and the median sagittal plane, the mesial 1 mm sagittal plane, and the distal 1 mm sagittal plane were also measured and recorded as the thickness of the labial bone wall of the implant (W2). The difference between the two was the size of the jumping space (HDD ═ W2 – W1) (Figure 4). The jumping space was measured after immediate implant placement. The measurement site was at the shoulder of the implant.

### Registration of digital models

**Figure 5.**
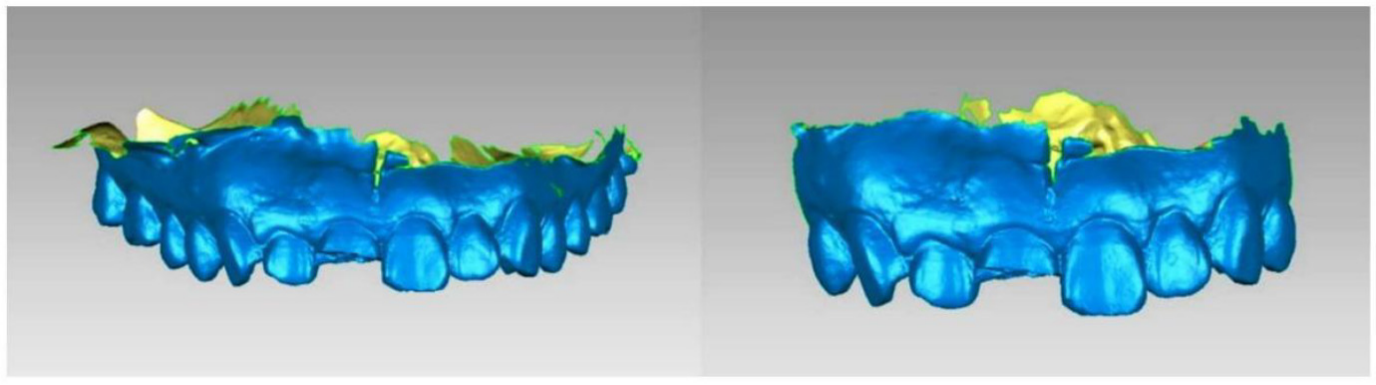
**The digital model of maxillary complete dentition.** The model was obtained and trimmed to retain the area between the bilateral first premolars.

**Figure 6.**
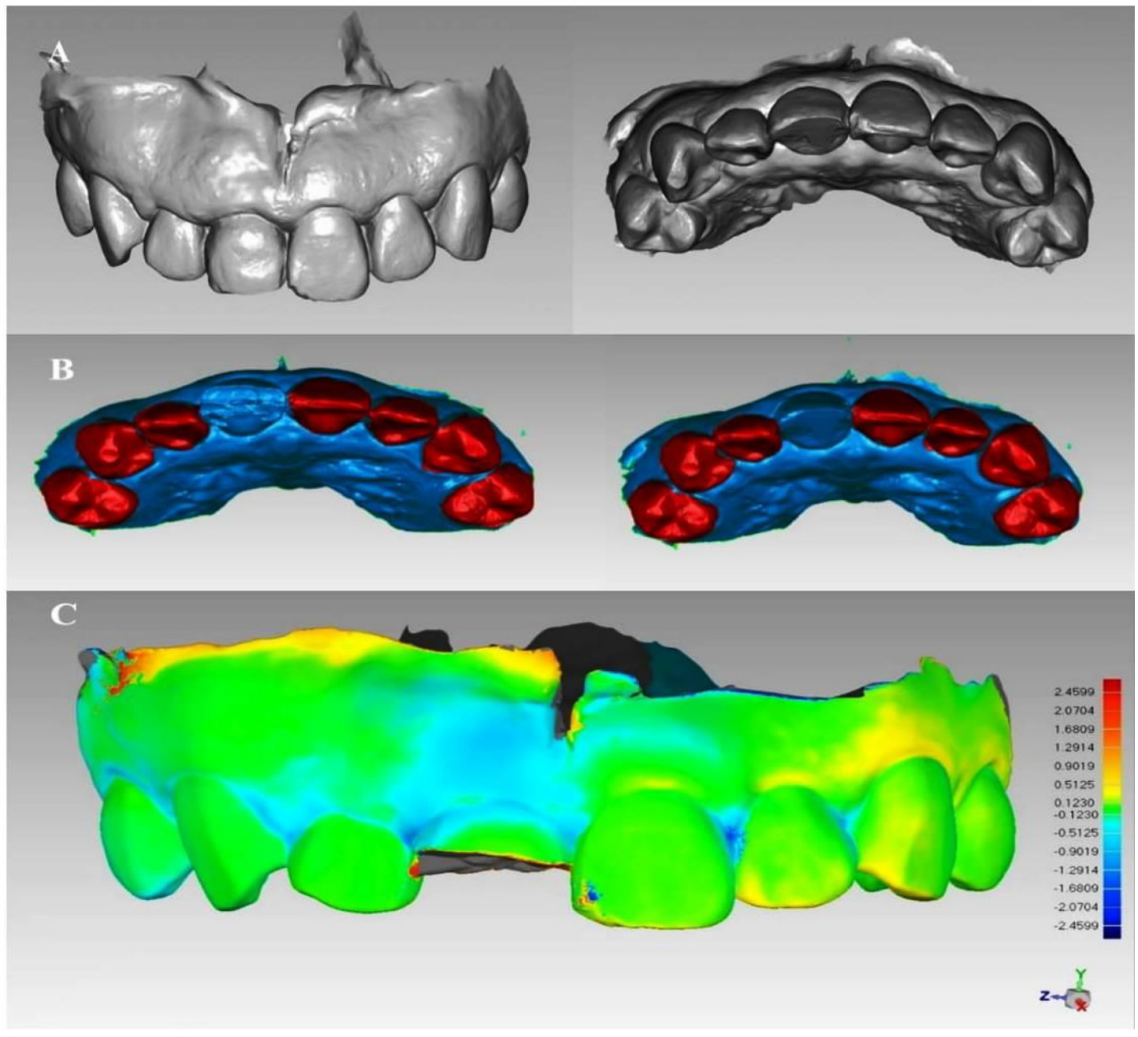
(A) The oral scan model in the sixth month after the operation; (B) The similar surface (red area) was selected by hand according to the rest of the teeth to overlap between the two models; (C) Sixth month after the operation, the model was superimposed with the preoperative model, and the shape changes of the labial soft tissue at the implant site were shown by chromatography. The light blue area visible on the 11-lip side was soft tissue collapse to the palatal side.

To facilitate model registration, the acquired digital model file was imported into Geomagic Studio 2013 with the retention of the area between the bilateral first premolars and the deletion of the rest (Figure 5). Intraoral scan documents at the first month, third month, and sixth month after operation were each registered based on the preoperative intraoral scan model. The registration method included: first, the use of “N-point alignment” in the “alignment” function of Geomagic Studio to select the cusp points with obvious characteristics of the two models to be registered and to ensure the consistency of the selected points; second, performing the “alignment” function; third, the use of the “best-fit alignment” for correction (Figure 6B). The two superimposed models were analyzed by using the “analysis” function, and the changing areas of soft tissue around the implantation site were marked by 15-color chromatography (Figure 6C) [32].

#### Establishment of a coordinate system

**Figure 7.**
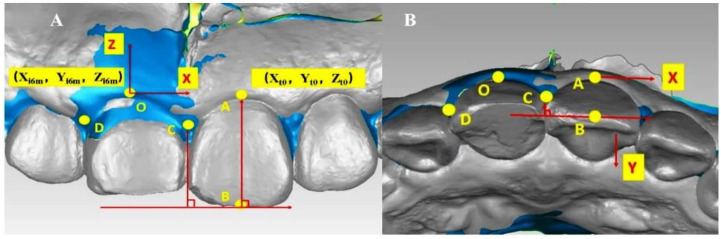
**The establishment of the coordinate system.** (A) Positive view, at the sixth month after the operation and the superposition of the preoperative model, it can be observed that the blue area is the 11-change area of soft tissue contour after immediate implantation and immediate repair. The coordinate origin O is the most coronal of 11-gingival margin, point A is the midpoint of 21-gingival margin, and point B is the midpoint of 21-cutting end, which is determined as the *Z*-axis by point O parallel to the AB line. The vertical line connecting AB through point B is parallel to the horizontal line passing through point O and is determined as the *X*-axis. Mesial gingival papilla and distal gingival papilla are marked as point C and point D, respectively; (B) Point C is perpendicular to the tangent lip and palate, which is determined as the *Y*-axis and perpendicular to the *X*-axis.

Digital models obtained at the first month, third month, and sixth month after operation were superimposed or registered with the preoperative model. The most coronal side of the implant site’s gingival margin was marked as the coordinate origin O. The midpoint of the gingival margin and the crown’s incisal edge of the homonym teeth on the contralateral side were taken as point A and point B, respectively. The direction of the A and B connection was determined as the *Z*-axis, which was parallel to the vertical line at point O. The tangent of the incisal edge of the crown was made by point B, which was perpendicular to the AB line and parallel to the horizontal line at point O. It was set as the *X*-axis, which was perpendicular to the *Z*-axis. The mesial gingival papilla was marked as point C and the distal gingival papilla was marked as point D. A vertical line was made to the incisal edge of the crown through point D in the occlusal surface, which was determined as the *Y*-axis. The *Y*-axis was perpendicular to the *X*-axis. Based on this, the *X*–*Y*–*Z* coordinate system was constructed (Figure 7).

#### 3D morphological reconstruction of soft tissue changes

The maximum critical value was set as 2.45 mm within the software after aligning and superimposing the postoperative scanning model with the preoperative scanning model. The maximum nominal and minimum nominal values were +0.123 mm and −0.123 mm, respectively, indicating that colors other than green will be displayed when the change was greater than ±0.123 mm. Therefore, it could be observed that the alignment part of the model was green, and the changing area of the soft tissue contour was light blue. The darker the blue color was, the greater the amount of change was (Figure 6C).

**Figure 8.**
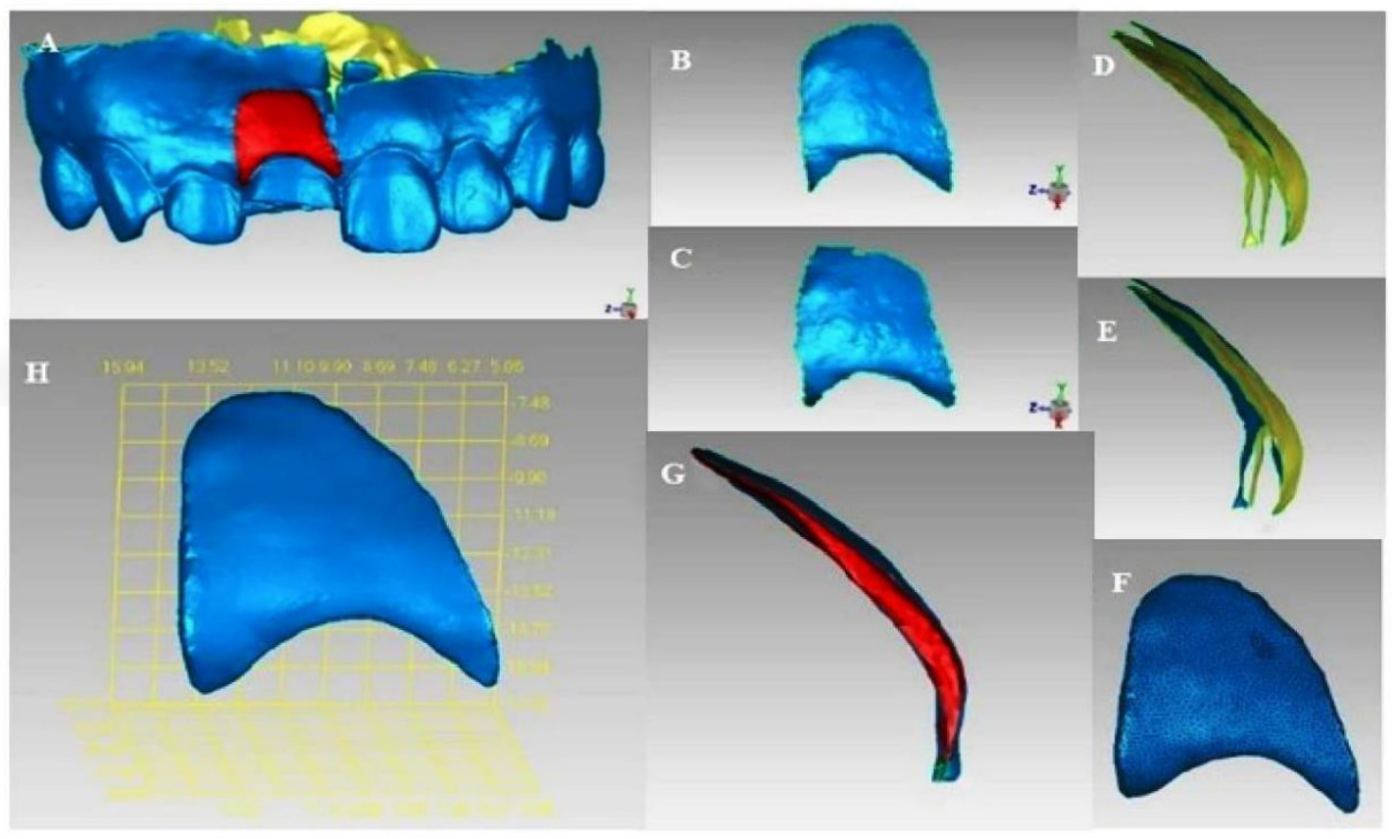
**Reconstruction of 3D morphology of soft tissue changes.** (A) Manually marking the changing area of the soft tissue in the implantation site; (B and C) Two local surfaces were obtained after trimming according to the 3D superimposed chromatographic display of the two models; (D) The two local surfaces were re-imported into the software, blue as the outer surface and yellow as the inner surface; (E) The local surface at the sixth month was flipped after the operation to form a potential gap between the two local surfaces; (F and G) The open area of this space was filled to obtain a closed space; (H) Reconstruction of a 3D shape of soft tissue changes.

According to the chromatographic display, the peri-implant soft tissue change area was manually marked (Figure 8A). The green display part and the frenulum mucosa were removed and the light blue display part was retained, thus the two local surfaces of the changing area were obtained (Figure 8B and 8C). The local surface in the sixth month after operation was selected, and the “flip normal” functions in Geomagic Studio 2013 software were used to flip it. A space inside the two local surfaces could be observed, but the space was not completely closed (Figure 8D and 8E). After that, the “fill” function in the software was filled to fill the edge gap in a way that matches the curvature of the surrounding mesh, thus forming an airtight space (Figure 8F). The reconstructed 3D model was the contour volume change of the soft tissue around the implantation site (Figure 8G and 8H).

### Measurement and analysis of 3D shape

#### Volume measurement of soft tissue changes

**Figure 9.**
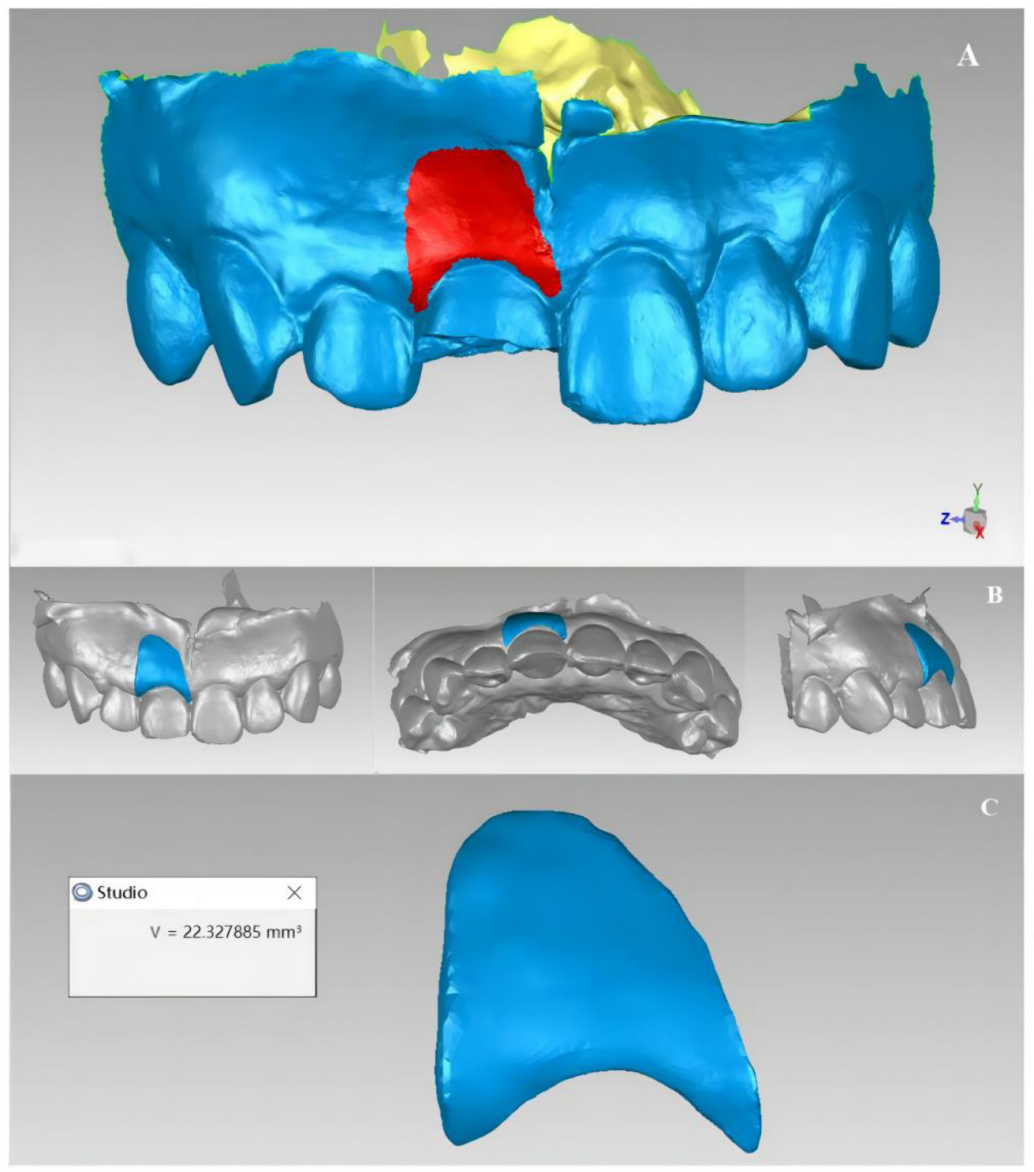
(A) Calculation of the surface area of the selected area (shown in red), S (mm^2^); (B) Changes in the 3D construction of the soft tissue (blue display); (C) Calculation of the volume of soft tissue changes, V (mm^3^).

All measurements were made on the superimposed model STL file by the same researcher. For the labial side of the implant region, the effective area of examination was determined based on the superimposed chromatographic display difference. The “calculation” function in the Geomagic Studio “Analysis” command was used to calculate the surface areas S1 (mm^2^) and S2 (mm^2^) of the soft tissue changes areas selected by the two models and the volume of the reconstructed soft tissue changes ΔV (mm^3^) (Figure 9). Since the size of the volume was mainly determined by the selected region’s size, the data were used as the average distance (MD: Δd [mm]). The calculation formula was Δd (mm) ═ ΔV(mm^3^)/ΔS(mm^2^); ΔS=$\frac{S_{1}+S_{2}}{2}$ sagittal plane.

#### Linear measurement of soft tissue changes

**Figure 10.**
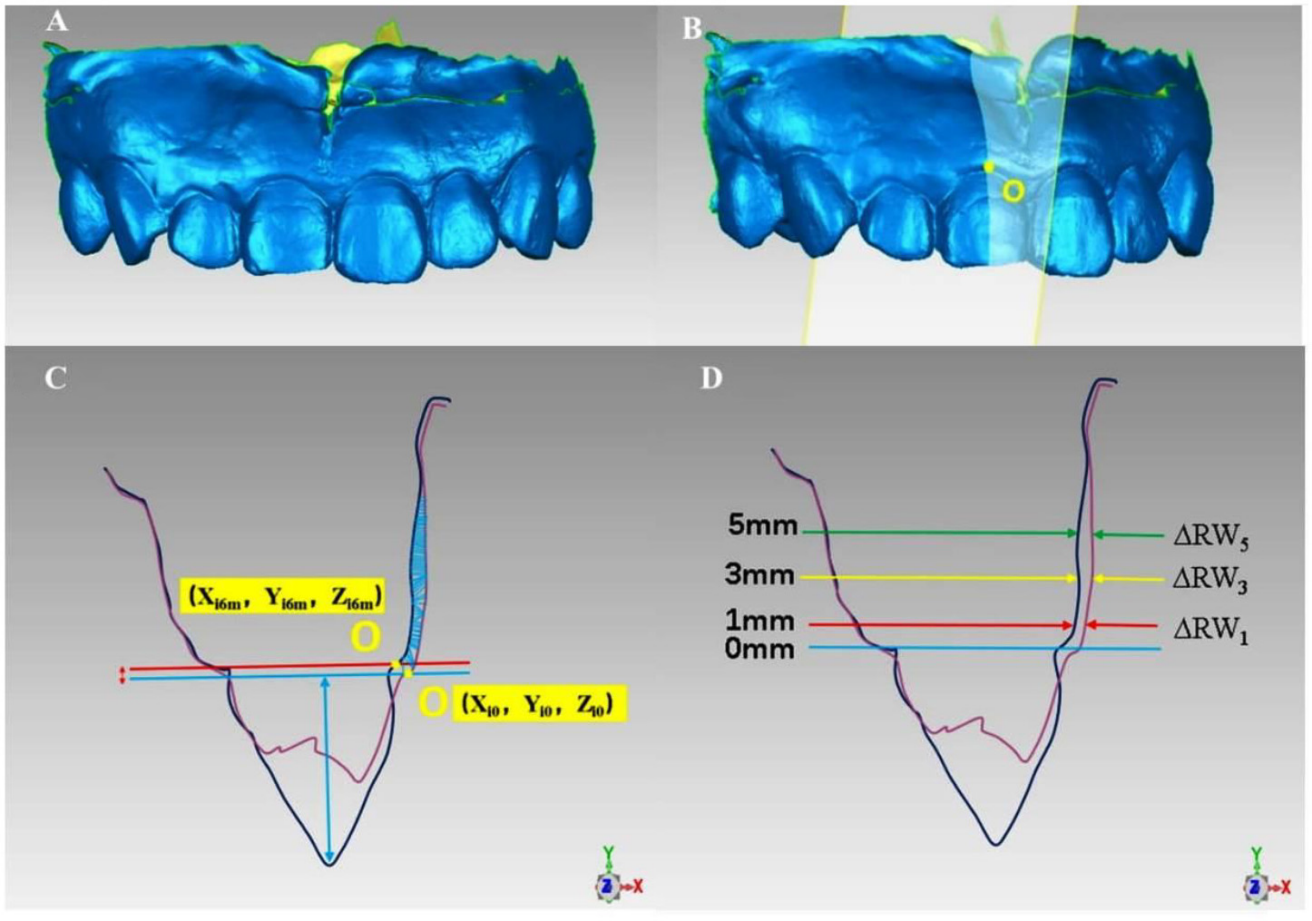
**Linear measurement of soft tissue changes.** (A) Preoperative STL file and follow-up STL file were aligned and merged into a whole; (B) Cross-section was created by point O and parallel to the implant; (C) Cross-sectional view, the change of gingival marginal level was evaluated by baseline phase and clinical crown height during postoperative follow-up. The purple line represents the outline of the tissue before the operation; the dark blue line indicates the outline of the tissue at the 6th month after the 11th month operation; the blue area indicates the change of the tissue at the sixth month after immediate implantation; the red double arrow represents the retraction of the labial gingival margin; the blue double arrow represents the clinical crown height; (D) The width of the alveolar ridge of 1 mm, 3 mm, and 5 mm (ΔRW1, ΔRW3, and ΔRW5) was measured at the same cross-section. STL: Standard Tessellation Language.

After the preoperative STL model was superimposed with the postoperative follow-up models, the “Boolean operation” function in the software was used to integrate the two aligned models (Figure 10A). The merged model was imported into Geomagic Qualify 2013 and the curve function section in tools was used to create the section. According to the establishment of the coordinate system mentioned earlier, the cross-section was selected through point O and parallel to the implant’s long axis (Figure 10B). The thickness of the lip and palate of the peri-implant tissue had been determined via this cross-sectional view. The changes in alveolar ridge width (RW) were measured at three levels below the edge of the gingival mucosa: submarginal 1 mm (ΔRW1), submarginal 3 mm (ΔRW3), and submarginal 5 mm (ΔRW5). All the measurements reflected the change of tissue thickness around the implant in the median.

**Figure 11.**
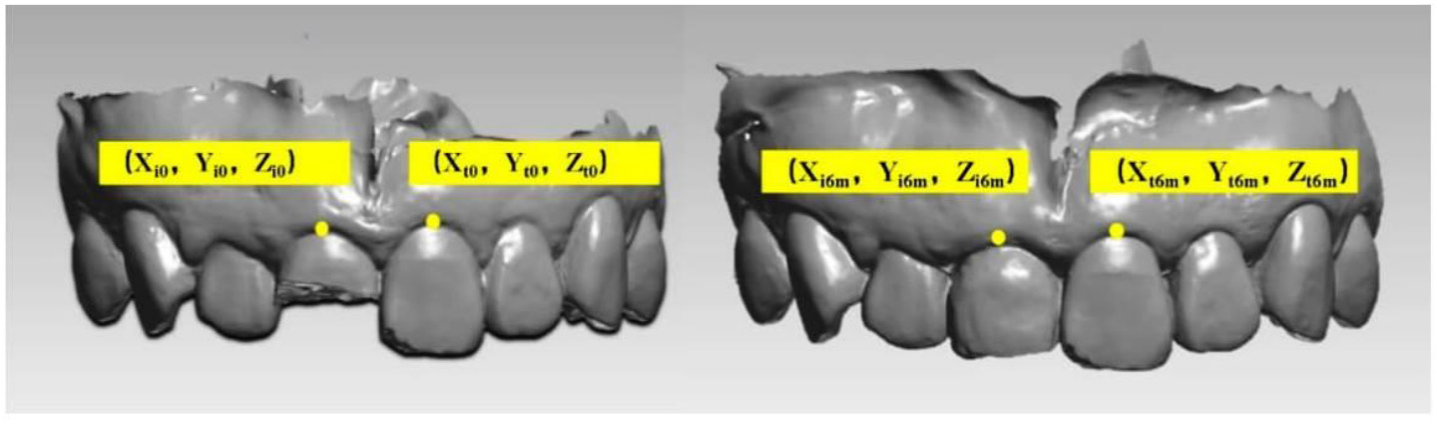
Using a coordinate system to mark both the vertex of the gingival margin and the adjacent gingival margin of the implant site before operation (baseline) and during follow-up.

The labial mucosal level (ML) of the implant at the baseline depends on the gingival vertex O (Zi0) on the implant’s labial side and the gingival vertex A (Zt0) on the labial side of the contralateral homonymous tooth: ML0 ═ Zi0 − Zt0. Similarly, the ML in the first, third, and sixth month after surgery was ML1m ═ Zi1m − Zt1m; ML3m ═ Zi3m − Zt3m; and ML6m ═ Zi6m − Zt6m, respectively. A positive value of ML indicates that the gingival margin of the implant site was located at the root of the contralateral tooth of the same name, and vice versa. Based on this, the change in the level of the gingival margin was ΔML ═ Zi6m − Zi0 (Figure 10C). The change in gingival margin level in each period was ΔML1m ═ ML1m − ML0 in the first month after the operation, ΔML3m ═ ML3m − ML0 in the third month after the operation, and ΔML6m ═ ML6m − ML0 in the sixth month after the operation. A positive value of ΔML indicates a recession of the implant site’s gingival margin, while a negative value indicates vice versa (Figure 11).

#### Esthetic evaluation of labial gingival soft tissue of implant

At T3, standardized digital images (Nikon D90 SLR camera) were used to evaluate the pink esthetic score (PES) of the implanting area based on the evaluation requirements suggested by Botticelli et al. [22]. Digital images were taken by the same doctor using the same camera. Standardization means that the physician took pictures with reference to oral photography books, such as standard shooting angles and image proportions. PES consists of seven indices: mesial papilla (0–2), distal papilla (0–2), alveolar process deficiency (0–2), soft-tissue level, contour, color, and texture (0–2). The maximum achievable score was 14. The PES scores of each group after permanent restoration were measured in the sixth month after the operation.

### Subjective satisfaction of patients

The evaluation of patient satisfaction was carried out at T3, and the overall satisfaction was evaluated by the patient self-evaluation questionnaire [33].

The satisfaction of color and shape was evaluated by a numerical visual analogue scale which ranged from 0 (“very dissatisfied”) to 10 (“very satisfied”).

### Ethical statement

Research experiments conducted in this study with humans were approved by the Ethical Committee and responsible authorities of Shanxi Medical University School and Hospital of Stomatology (No. SXMYU-2021-0301), following all guidelines, regulations, legal and ethical standards as required for humans.

### Statistical analysis

Data were entered into Excel (Microsoft Company, USA) after measurement. SPSS 24.0 was used for statistical analysis, and all values are expressed as X ± standard deviation. When the data met all the normality and homogeneity of variance criteria, a single group of repeated measurement analysis of variance was performed for the determination of differences between the groups. To test for normality of distribution, the Shapiro–Wilk test was performed. Nonparametric test (Kruskal–Wallis test) was conducted to analyze the changes in soft tissue and PES between different groups when the quantitative data failed to meet the requirements of normality and homogeneity of variance. The Pearson correlation coefficient was conducted to study the correlation between the size of jumping space and the volume and linear change of soft tissue contour d uring operation, and the scatter plot was used to represent it. *P* value < 0.05 showed statistically significant difference.

## Results

### Patients and implants

A total of 32 patients (13 males and 19 females; median age: 31 years) with 32 implants (Nobel Active 24, Ankylos 8) in the upper anterior region treated with the IIPR technique were included. The sex distribution, age, implant site, cause of tooth extraction, and implant type of the patients are shown in Table 1. The size of the labial jumping space was measured by CBCT from the sagittal plane of the three adjacent 1 mm gaps, with the average value 2.61 ± 0.60 mm. All implants achieved osseointegration during the follow-up period. Five patients (four males and one female) had temporary crown loosening as a result of the loosening of the central screw in the temporary abutment. The follow-up was completed after using a screwdriver to tighten with appropriate torque. After six months of follow-up, three patients (two males and one female) continued to use the new temporary crown for gingival shaping because of the lack of soft tissue esthetics at the implantation site. Table 2 shows the normal distribution of data.

**Table 1:** Baseline characteristics of the study sample

	**Group A (*n* ═ 7)**	**Group B (*n* ═ 16)**	**Group C (*n* ═ 9)**	**Total (*n* ═ 32)**
*Jumping spaces (mm)*				
Mean ± SD	1.84 ± 0.15	2.58 ± 0.21	3.36 ± 0.18	2.62 ± 0.19
Range	1.62–1.92	2.23–2.94	3.11–3.52	1.62–3.52
*Sex*				
Male	3 (43%)	6 (38%)	4 (44%)	13 (41%)
Female	4 (56%)	10 (62%)	5 (66%)	19 (59%)
*Age (years)*				
Mean ± SD	34.3 ± 5.5	39.4 ± 9.3	33.4 ± 9.2	36.2 ± 7.5
Median	30	39	34	31
Range	24–39	20–53	21–47	20–53
*Implant site*				
Central incisor	3 (43%)	9 (56%)	4 (44%)	16 (50%)
Lateral incisor	4 (57%)	5 (31%)	3 (33%)	12 (38%)
Canine	0	2 (13%)	2 (23%)	4 (12%)
*Cause of tooth extraction*				
Injury	4 (57%)	10 (62%)	5 (56%)	19 (59%)
Caries/pulp disease	2 (28%)	4 (25%)	2 (22%)	8 (25%)
Other	1 (15%)	2 (13%)	2 (22%)	5 (16%)
*Implant type*				
Nobel active	5 (71%)	12 (75%)	7 (78%)	24 (75%)
Ankylos	2 (29%)	4 (25%)	2 (22%)	8 (25%)

**Table 2:** Shapiro–Wilk test for testing the normal distribution

**Statistic**	**df**	***P* value**
0.745	28	0.001
0.698	28	0.000
0.785	28	0.001

df: Degrees of freedom.

### Measurement and analysis of 3D morphology of soft tissue around the implant

#### Qualitative analysis

For six months of follow-up, all patients showed a decrease in the volume of the labial profile, which signifies a decrease in the thickness of the alveolar ridge and different degrees of recession in the gingival margin.

#### Quantitative analysis

**Figure 12.**
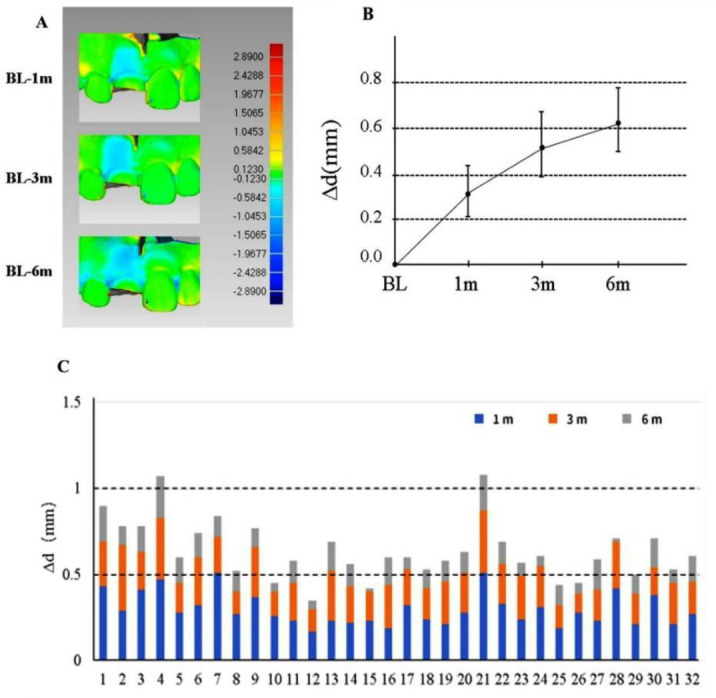
**Changes in average thickness of soft tissue contours (Δd) in 32 patients.** (A) The changes of labial soft tissue contour at the implant site at the first month, third month, and sixth month after the operation; (B) The average thickness changes from baseline to the sixth month after the operation; (C) The average thickness changes of soft tissue contour in 32 patients. BL: Baseline.

For 6 months of follow-up, the average change of gingival mucosa level on the labial side (ΔML) was 0.42 ± 0.12 mm, and statistically significant differences had been obtained in the first month, third month, and sixth month after the operation (*P* ═ 0.031). A significant difference has been obtained between ΔML1m and ΔML6m (*P* ═ 0.016), but no significant difference has been obtained between ΔML1m with ΔML3m (*P* ═ 0.462) and between ΔML3m with ΔML6m (*P* ═ 0.231). The average thickness change of the labial soft tissue profile at the implant site was 0.62 ± 0.15 mm, and statistically significant differences were found in the first month, third month, and sixth month after the operation (*P* < 0.001). There was statistical significance between Δd1m and Δd3m and between Δd1m and Δd6m (*P* < 0.001), but no statistical significance was found between Δd3m and Δd6m (*P* ═ 0.462). The average linear measurement of 1 mm from the gingival margin was 0.29 ± 0.09 mm, and there was no significant change in the first month, third month, and sixth month after the operation (*P* ═ 0.078). The average linear measurement of 3 mm from the gingival margin was 0.38 ± 0.11 mm, and there was no significant change in the first month, third month, or sixth month after the operation (*P* ═ 0.054). The average linear measurement of 5 mm from the gingival margin was 0.50 ± 0.14 mm. There were significant changes in the first month, third month, and sixth month after the operation (*P* ═ 0.029) and significant differences between T1 and T2 (*P* ═ 0.023) and T1 and T3 (*P* ═ 0.016). There was no statistical difference between T1 and T2 (*P* ═ 0.143). All subjects showed good PES values during the 6 months of follow-up with an average of 11.09 (Figure 12 and Table 3).

**Table 3:** Changes of each measurement index with time

	**Month 1 after the operation (T1)**	**Month 3 after the operation (T2)**	**Month 6 after the operation (T3)**	***P* value**	**Pairwise comparison**
Δ ML (mm)	0.24 ± 0.12	0.31 ± 0.21	0.42 ± 0.12	0.031*	T1 vs T2; *P* ═ 0.462 T1 vs T3; *P* ═ 0.016* T2 vs T3; *P* ═ 0.231
Δd (mm)	0.32 ± 0.12	0.53 ± 0.14	0.62 ± 0.15	<0.001*	T1 vs T2; *P* <0.0 01* T1 vs T3; *P* <0.0 01* T2 vs T3; *P* ═ 0.462
Δd ≤ 0.5 mm	30 (94%)	27 (84%)	6 (19%)	–	–
0.5 mm< Δd ≤1 mm	2 (6%)	15 (16%)	24 (75%)	–	–
Δd>1 mm	0	0	2 (6%)	–	–
ΔRW1 (mm)	0.15 ± 0.08	0.25 ± 0.14	0.29 ± 0.09	0.078	–
ΔRW3 (mm)	0.22 ± 0.12	0.31 ± 0.23	0.38 ± 0.11	0.054	–
ΔRW5 (mm)	0.24 ± 0.22	0.41 ± 0.15	0.50 ± 0.14	0.029*	T1 vs T2; *P* ═ 0.023* T1 vs T3; *P* ═ 0.016* T1 vs T3; *P* ═ 0.143
PES	11.24 ± 0.87	11.21 ± 0.93	11.09 ± 0.99	0.284	–

*Statistically significant. PES: Pink esthetic score; ΔML: Average change in the labial mucosal level; ΔRW1: Average change of alveolar ridge width at 1 mm level under the gingival margin; ΔRW3: Average change of alveolar ridge width at 3 mm level under the gingival margin; ΔRW5: Average change of alveolar ridge width at 5 mm level under the gingival margin; Δd: Average thickness change.

### Comparative analysis of 3D morphology of soft tissue around implants in three groups

Table 4 shows the changes of gingival mucosa levels in three groups with different jumping spaces six months after the surgery. The change of gingival mucosa level in group A (0 ∼ 2 mm) was 0.45 ± 0.11 mm, in group B (2 ∼ 3 mm) was 0.40 ± 0.12 mm, and in group C (≥ 3 mm) was 0.35 ± 0.11 mm. The result of Kruskal–Wallis test was 0.315. The test level was alpha ═ 0.05, and it can be considered that no significant difference result was obtained in the gingival mucosa level after different jumping spaces.

**Table 4:** Changes of gingival mucosa level in the three groups six months after operation

**Group**	**ΔML (mm) 6 months after the operation**	***P* value**	**Pairwise comparison**
A (HDD 0 ∼ 2 mm)	0.45 ± 0.11	0.315	–
B (HDD 2 ∼ 3 mm)	0.40 ± 0.12		–
C (HDD ≥ 3 mm)	0.35 ± 0.11		–

ΔML: Average change in the labial mucosal level; HDD: Horizontal defect dimension.

Table 5 shows the changes of the average thickness of contour volume in three groups of different jumping spaces six months after operation. The average thickness change in group A was 0.77 ± 0.16 mm, in group B was 0.63 ± 0.17 mm, and in group C was 0.54 ± 0.11 mm. The result of the Kruskal–Wallis test was 0.02. The test level was alpha ═ 0.05, and it can be considered that there were differences in the average thickness of the volume profile between the three groups with different jumping spaces. After pairwise comparison, no significant difference was obtained in the average thickness change between groups A and B (*P* ═ 0.179) and between groups B and C (*P* ═ 0.554). The average thickness change between groups A and C showed statistically significant result (*P* ═ 0.015).

**Table 5:** Changes of the average thickness of soft tissue contour volume in three groups six months after operation

**Group**	**Δd (mm) 6 months after the operation**	***P* value**	**Pairwise comparison**
A (HDD 0 ∼ 2 mm)	0.77 ± 0.16	0.02*	A vs B; *P* ═ 0.179
B (HDD 2 ∼ 3 mm)	0.63 ± 0.17		B vs C; *P* ═ 0.544
C (HDD ≥ 3 mm)	0.54 ± 0.11		A vs C; *P* ═ 0.015*

*Statistically significant. HDD: Horizontal defect dimension; Δd: Average thickness change.

Table 6 shows the linear changes of the profile six months after operation in three groups of different jumping spaces. The average linear variation of alveolar RW at the level of subgingival 1 mm (ΔRW1) was 0.35 mm (0.26–0.49 mm) in group A, 0.29 mm (0.12–0.52 mm) in group B, and 0.24 mm (0.16–0.32 mm) in group C. The result of the Kruskal–Wallis test was 0.066. The test level was alpha ═ 0.05, and it can be considered that there was no statistical significance in the linear change of contour profile with different jumping spaces.

The average linear change of alveolar RW at subgingival margin 3 mm level (ΔRW3) was 0.47 mm (0.42–0.55 mm) in group A, 0.38 mm (0.21–0.59 mm) in group B, and 0.31 mm (0.18–0.47 mm) in group C. The result of the Kruskal–Wallis test was 0.023. The test level was alpha ═ 0.05, and it can be considered that the linear change of contour profile after different jumping spaces has statistical significance. After pairwise comparison, there was no significant linear change between groups A and B (*P* ═ 0.165) and between groups B and C (*P* ═ 0.661). The linear change between groups A and C was statistically significant (*P* ═ 0.018).

**Figure 13.**
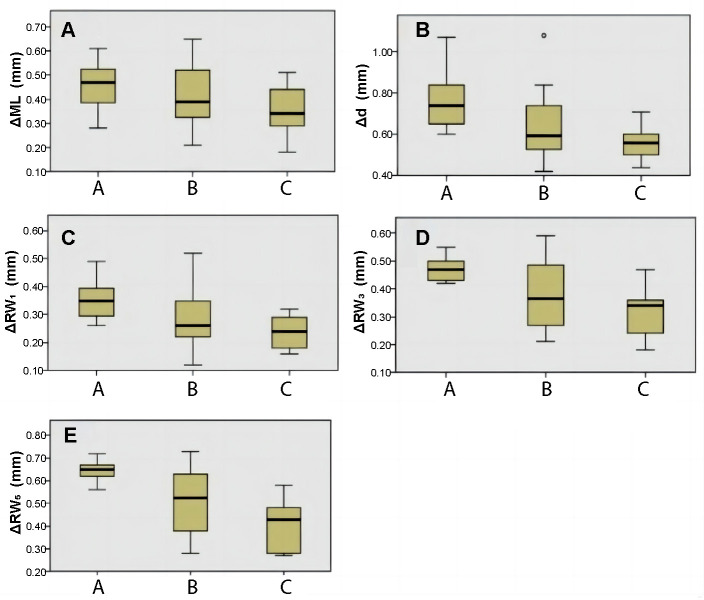
**Measurement indices changes in three groups.** (A) Changes in the level of gingival mucosa (ΔML); (B) The variation of the average thickness of three groups of contour volumes (Δd); (C) Average change of alveolar ridge width at 1 mm level under the gingival margin (ΔRW1); (D) Average change of alveolar ridge width at 3 mm level under the gingival margin (ΔRW3); (E) Average change of alveolar ridge width at 5 mm level under the gingival margin (ΔRW5).

The average linear change of alveolar RW at subgingival margin 5 mm level (ΔRW5) was 0.64 mm (0.56–0.72 mm) in group A, 0.50 mm (0.28–0.73 mm) in group B, and 0.40 mm (0.27–0.58 mm) in group C. The result of the Kruskal–Wallis test was 0.005. The test level was alpha ═ 0.05, and it can be considered that there were differences in the linear changes of contour profiles among the three groups with different jumping spaces. After pairwise comparison, there was no significant linear change between groups A and B (*P* ═ 0.090) and between groups B and C (*P* ═ 0.343). The linear change between groups A and C was statistically significant (*P* ═ 0.003) (Figure 13).

### Results of correlation analysis

Pearson correlation analysis had been carried out to evaluate the relationship between the different jumping spaces with the changes of gingival mucosa level and the average thickness of peri-implant contour volume (Table 7). The Pearson’s correlation cutoff value was 0.2–0.39 for weak, 0.40–0.59 for moderate, 0.6–0.79 for strong correlation, and 0.8–1 for very strong correlation. There was a linear relationship between these variables, which was a moderate negative correlation of *r* ═ −0.353 (*P* ═ 0.048) and *r* ═ 0.632 (*P* < 0.001), respectively.

There was a non-linear relationship between the PES and the different intraoperative jumping spaces (*r* ═ −0.292, *P* ═ 0.105).

Correlation analysis did not find that there was a linear correlation between the alveolar RW change at the level of subgingival 1 mm and the different jumping spaces during the operation (*r* ═ −0.383, *P* ═ 0.066).

**Figure 14.**
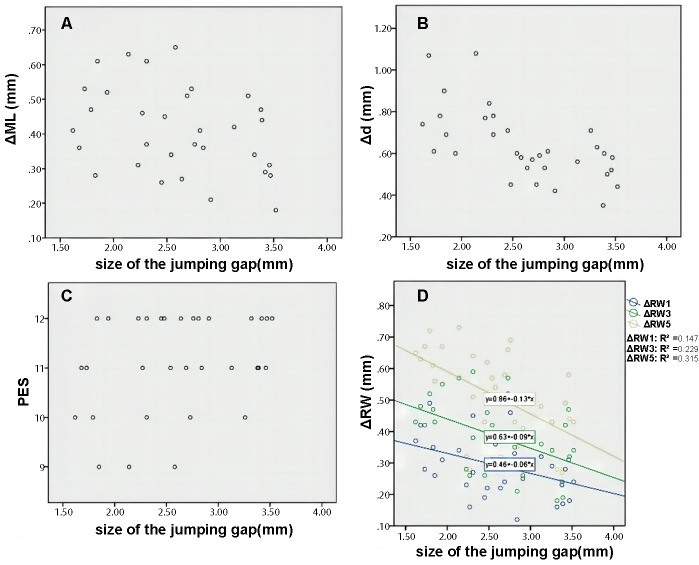
The scatter plots showing the relationship between the size of jumping space and (A) the level of labial gingival mucosa (ΔML); (B) the average thickness of labial profile; (C) pink esthetic score (PES); (D) the changes in alveolar ridge width at 1 mm, 3 mm, and 5 mm levels under the gingival margin (ΔRW1, ΔRW3, and ΔRW5, respectively).

Based on the correlation analysis, there was a linear correlation between the alveolar RW change at the level of subgingival 3 mm and 5 mm with different jumping spaces during the surgery at *r* ═ −0.479 (*P* ═ 0.023) and *r* ═ −0.561 (*P* ═ 0.005), respectively (Figure 14).

**Table 6:** Linear changes of soft tissue profile in three groups six months after operation

	**Group**	**ΔRW (mm) 6 months after the operation**	***P* value**	**Pairwise comparison**
ΔRW1 (mm)	A (HDD 0 ∼ 2 mm)	0.35 ± 0.08	0.066	
	B (HDD 2 ∼ 3 mm)	0.29 ± 0.11		–
	C (HDD ≥ 3 mm)	0.24 ± 0.06		–
ΔRW3 (mm)	A (HDD 0 ∼ 2 mm)	0.47 ± 0.04	0.023*	A vs B; *P* ═ 0.165
	B (HDD 2 ∼ 3 mm)	0.38 ± 0.12		B vs C; *P* ═ 0.661
	C (HDD ≥ 3 mm)	0.31 ± 0.09		A vs C; *P* ═ 0.018*
ΔRW5 (mm)	A (HDD 0 ∼ 2 mm)	0.64 ± 0.05	0.005*	A vs B; *P* ═ 0.090
	B (HDD 2 ∼ 3 mm)	0.50 ± 0.14		B vs C; *P* ═ 0.343
	C (HDD ≥ 3 mm)	0.40 ± 0.10		A vs C; *P* ═ 0.003*

*Statistically significant. ΔRW1: Average change of alveolar ridge width at 1 mm level under the gingival margin; ΔRW3: Average change of alveolar ridge width at 3 mm level under the gingival margin; ΔRW5: Average change of alveolar ridge width at 5 mm level under the gingival margin; HDD: Horizontal defect dimension.

### Results of patient satisfaction

Average score of overall satisfaction was 8.38 ± 2.44 for group A, 8.51 ± 2.06 for group B, and 8.52 ± 1.76 for group C (Table 8). No significant difference result was obtained between the three groups (*P* ═ 0.862). There were similar scores on the satisfaction of color and appearance of peri-implant mucosa between the three groups, however, no significant difference was found between the three groups (*P* ═ 0.702, *P* ═ 0.814).

**Table 7:** Correlation between intraoperative jumping space size and soft tissue/esthetic changes

**Variable**	**Pearson correlation coefficient**	***P* value**
ΔML	−0.353	0.048*
Δd	−0.632	<0.001*
PES	−0.292	0.105
ΔRW1	−0.383	0.066*
ΔRW3	−0.479	0.023*
ΔRW5	−0.561	0.005*

*Statistically significant. PES: Pink esthetic score; ΔML: Average change in the labial mucosal level; ΔRW1: Average change of alveolar ridge width at 1 mm level under the gingival margin; ΔRW3: Average change of alveolar ridge width at 3 mm level under the gingival margin; ΔRW5: Average change of alveolar ridge width at 5 mm level under the gingival margin; Δd: Average thickness change.

**Table 8:** Overall satisfaction with soft tissue esthetic effect at month three after operation

**Soft tissue esthetic effect**	**Group A**	**Group B**	**Group C**	***P* value**
Color of peri-implant mucosa	8.42 ± 2.06	8.53 ± 1.73	8.58 ± 1.56	0.702
Shape of peri-implant mucosa	8.15 ± 2.27	8.33 ± 2.13	8.38 ± 2.38	0.814
Total score of overall satisfaction	8.38 ± 2.44	8.51 ± 2.06	8.52 ± 1.76	0.862

## Discussion

Accurate and precise measurement of soft tissue is the basis for the evaluation of changes of soft tissue after IIPR. Previous protocol mostly consists of the preparation of an impression perfusion model, manual measurement of the model, and analysis of the changes in soft tissue [34]. The technique is easily affected by many factors, such as the shrinkage of impression material, the accuracy of mold taking, the expansion of gypsum material, etc. Therefore, its accuracy cannot be guaranteed and the measurement and analysis method of the gypsum model is relatively simple. Hence, some scholars utilize digital light scanning evaluation methods to measure the volume changes of oral soft tissue over time, enabling the longitudinal measurement and multiple quantifications of soft tissue volume after implantation. They can provide accurate, non-invasive, and visually intuitive observation for diagnosis, treatment planning, and treatment regimen evaluation. Most importantly, the adhibition of digital technology to evaluate treatment results and visualize the increase or decrease of postoperative total volume can be used to prove not only the statistical significance of different treatment options but also their clinical significance [34]. In this experiment, digital scanning data were used to evaluate the correlation between the changes in the soft tissue around the implant and different jumping spaces during the operation and showed that the contoured volume of labial soft tissue changed continuously after IIPR and reached relative stability in the sixth month after the operation, regardless of the size of the jumping space.

The change of labial soft tissue after IIPR may result from the remodeling of the alveolar bone below it and causes the changes in size of the alveolar crest. In a prospective clinical study, it was found that the alveolar ridge was significantly absorbed in the first three months, and the absorption of the buccal bone plate was more significant compared to the palatal side [35]. Araújo and Lindhe [36] found that the coronal area of the labial bone plate is almost entirely composed of fasciculate bone which is bound to be absorbed due to the disappearance of periodontal ligament after tooth extraction. This leads to the change of labial soft tissue, and this change cannot be avoided completely. The clinical trial of Sanz et al. [25] showed that the horizontal width of the alveolar ridge reduced by 1.10 mm (CI 0.90–1.30) after immediate implant placement. The measurement results of this experiment showed that the average thickness changes of the soft tissue profile after IIPR was 0.62 ± 0.15 mm. This may be due to the strict inclusion criteria of this study, the low replacement rate of bone substitute materials implanted in the jumping space, and the temporary restoration with screw retention used to maintain the gingival tissue after operation that made the difference. Arora et al. [37] reported that the change of horizontal profile was 0.56 ± 0.48 mm after 12 months, indicating that the above factors were beneficial in maintaining the peri-implant tissue.

The horizontal change of gingival mucosa after IIP results from horizontal and vertical bone resorption in the alveolar bone’s labial plate, which is almost completely composed of bundled bone. Some studies have confirmed that IIPR may cause the retraction of the marginal mucosa around the implant. Several factors that may affect the frequency and degree of marginal mucosal recession have been reported, including the gingival biotype, the temporary restoration application after IIP, the labial bone plate’s thickness, the implant’s 3D position in the alveolar socket, and the implantation of bone substitute in the jumping space [32] and flap surgery that destroys the labial bone plate’s blood supply. Therefore, some scholars suggest that IIP should be carried out without flap surgery or with a minimum one, which can not only maintain a good esthetic effect but also minimize the marginal recession of labial mucosa. Data from three studies were reported in the systematic review of Huang et al. [38], which contributed to the data regarding the changes in the level of soft tissue. The follow-up duration was 3, 6, and 12 months upon IIPR and majority of the soft tissue changes were found during the first 3 months [15]. The proximal and distal gingival papillae retreated 0.41 ± 0.32 mm and 0.34 ± 0.36 mm, respectively. Compared with the level before implantation, the mucosa around the buccal implant retreated to 0.43 ± 0.38 mm. After six months, the soft tissue became stable. After the first year, the mesial gingival papilla, the distal gingival papilla, and the buccal gingival margin retreated at 0.49 ± 0.31 mm, 0.36 ± 0.33 mm, and 0.51 ± 0.38 mm, respectively. The results of this study were similar to the gingival mucosal recession 6 months after the operation, which was 0.42 ± 0.12 mm. No significant difference was found between the three groups. This was probably caused by the use of non-flap surgery, low replacement rate of bone replacement materials, and temporary fixed prostheses.

According to the results of the correlation analysis between the three different jumping spaces grous and the change in gingival mucosa level, no significant relationship was found between them. The correlation analysis between the three different jumping spaces and the change in the mean thickness of the soft tissue contour volume around the implant was found to be negative, which means that the amount of change in the soft tissue contour volume may decrease as the jumping space increases. Some scholars have studied the effect of different jumping spaces on the changes of the alveolar ridge after IIP and carried out morphological measurement and analysis, which found that when compared with the buccal space of 1 mm, preserving the labial space of 2 mm and 3 mm can better maintain the soft and hard tissue around the implant [39]. Clinically, we can adjust the size of the labial jumping space by selecting the implant position in the alveolar sockets to reduce the change of labial soft tissue profile. In this experiment, the volume change of labial soft tissue was the smallest when the digital 3D model showed HDD > 3 mm. The reason is that the large gap between the implant and the labial bone wall increases the thickness of the alveolar bone formed on the labial side after the operation. The thicker the alveolar bone is, the more stable it is. It is also resistant to the remodeling and absorption of the labial alveolar bone. Therefore, during IIP, the labial side of the implant should be reserved as large as possible, and the bone substitute with a low replacement rate (DBBM) should be filled to form the best thickness of labial alveolar bone and soft tissue to maintain the profile of soft tissue around the implant.

The profile analysis of soft tissue contour indicated no significant relationship between the different jumping spaces during operation with the changes of alveolar ridge thickness at the level of subgingival 1 mm but there was a significant relationship with the subgingival margin 3 mm and 5 mm levels considering that the reason may be the application of temporary restoration. Flügge et al. [40] examined the soft tissue plasticity of temporary fixed restoration after IIP and found that in the esthetic area, the bone-level implant can be used to individualize the design of the emergence profile and the edge position of the crown. In this case, the temporary restoration adjustments and the soft tissue structure shaping around the implant, including the mucous membrane, the gingival papilla, the soft tissue edge of the neck, and the position of the final high point of the gingival margin. Therefore, it is recommended to use temporary fixed restoration after immediate implant placement in the esthetic area. In this study, the manufacture of the temporary restoration was close to the gingival profile of the original natural teeth to support the gingival tissue effectively, so that 1 mm under the gingival margin showed the least change due to the support of the temporary restoration. Due to the lack of rigid support in the subgingival 3 mm and 5 mm levels, the soft tissue profile changed greatly along with the remodeling and absorption of the labial alveolar bone.

However, these conclusions still need to be treated with caution as small subject size was included in this experiment. But the results of this study showed that as long as the appropriate cases are screened (complete extraction sockets and sufficient bone mass to ensure the initial stability of the implant), strict clinical treatment procedures (flapless surgery, keeping jumping space as large as possible on the labial side of the implant and placing a low replacement rate bone substitute in the gap, using a temporary fixed prosthesis to maintain gingival shape after operation), and correct implant position, IIPR technique can achieve good esthetic results. The profile of the soft tissue on the labial side of the implant can be effectively maintained. 

## Conclusion

Through the measurement and observation of the contoured volume of labial soft tissue in the upper anterior teeth of 32 patients for 6 months after IIPR, we concluded that:

1. There was a persistent reduction in volume of labial soft tissue for six months after IIPR, and the changes mainly occured in the first three months.
2. The gingival mucosa levels of patients with different jumping spaces had a small recession after operation, and the linear measurement of the soft tissue profile showed that the change of subgingival 5 mm level was the largest. There was a moderate negative correlation between the size of intraoperative jumping space and the average thickness of soft tissue volume profile, the level of gingival mucosa, and the linear change of submarginal 3 mm and 5 mm soft tissue profile. However, there was no significant correlation between the size of the intraoperative jumping space and the pink esthetic score with the linear change of submarginal 1 mm soft tissue profile. Therefore, enough jumping space should be set aside as far as possible during the IIP of the upper anterior teeth, which can reduce the contour changes of the soft tissue around the implant.
3. The IIPR procedure of upper anterior teeth can achieve a satisfactory esthetic effect, and the peri-implant soft tissue level can be well maintained.
